# Two-Way Development of the Genetic Model for Endometrial Tumorigenesis in Mice: Current and Future Perspectives

**DOI:** 10.3389/fgene.2021.798628

**Published:** 2021-12-09

**Authors:** Yoshiaki Maru, Yoshitaka Hippo

**Affiliations:** Department of Molecular Carcinogenesis, Chiba Cancer Center Research Institute, Chiba, Japan

**Keywords:** genetically engineered mouse, organoid, endometrial cancer, carcinogenesis, mouse model, immunodeficient mice

## Abstract

Endometrial cancer (EC) is the most common malignancy of the female reproductive tract worldwide. Although comprehensive genomic analyses of EC have already uncovered many recurrent genetic alterations and deregulated signaling pathways, its disease model has been limited in quantity and quality. Here, we review the current status of genetic models for EC in mice, which have been developed in two distinct ways at the level of organisms and cells. Accordingly, we first describe the *in vivo* model using genetic engineering. This approach has been applied to only a subset of genes, with a primary focus on *Pten* inactivation, given that *PTEN* is the most frequently altered gene in human EC. In these models, the tissue specificity in genetic engineering determined by the *Cre* transgenic line has been insufficient. Consequently, the molecular mechanisms underlying EC development remain poorly understood, and preclinical models are still limited in number. Recently, refined *Cre* transgenic mice have been created to address this issue. With highly specific gene recombination in the endometrial cell lineage, acceptable *in vivo* modeling of EC development is warranted using these *Cre* lines. Second, we illustrate an emerging cell-based model. This hybrid approach comprises *ex vivo* genetic engineering of organoids and *in vivo* tumor development in immunocompromised mice. Although only a few successful cases have been reported as proof of concept, this approach allows quick and comprehensive analysis, ensuring a high potential for reconstituting carcinogenesis. Hence, *ex vivo/in vivo* hybrid modeling of EC development and its comparison with corresponding *in vivo* models may dramatically accelerate EC research. Finally, we provide perspectives on future directions of EC modeling.

## Introduction

Endometrial cancer (EC) is the most common gynecological malignancy and the fourth most common cancer in developed countries (Yamagami et al., 2017; Siegel et al., 2020). Risk factors for EC include age, obesity, diabetes, and exposure to unopposed estrogen or tamoxifen. The 5-year overall survival rate is between 74 and 90% for patients with stage I or II EC according to the International Federation of Gynecology and Obstetrics (FIGO) staging, whereas that of patients with EC in stage IV EC is 20–26% (Morice et al., 2016). Patients with the following hereditary diseases are susceptible to EC development: Cowden’s disease, Peutz–Jeghers syndrome, and Lynch syndrome, which are caused by germline mutations in *PTEN* (Blumenthal and Dennis, 2008), *LKB1* (Hearle et al., 2006), and mismatch repair genes (Meyer et al., 2009), respectively.

According to the traditional classification, EC is roughly divided into two distinct subtypes based on clinical and pathological characteristics. Type I EC, comprising 80% of all ECs, is mainly associated with estrogen dependency, endometrial hyperplasia, and a favorable prognosis. The predominant histological subtype is endometrioid carcinoma, harboring mutations most frequently in *PTEN*, followed by *PIK3CA*, *KRAS*, *ARID1A*, and *CTNNB1*. Type II EC, comprising various minor subtypes including serous carcinoma, clear cell carcinoma, and carcinosarcoma (CS), is associated with estrogen independence, endometrial atrophy, and poor prognoses. Mutations in *TP53*, *PIK3CA*, *PPP2R1A*, and *ERBB2* amplification are among the most frequent genetic alterations (Kandoth et al., 2013; Murali et al., 2014). Based on comprehensive genomic profiling of EC, four molecular subtypes have been recently proposed: DNA polymerase epsilon (POLE) ultramutated type, microsatellite instability (MSI) hypermutated type, copy number low type, and copy number high type. Patients with the first two subtypes have a significantly better prognosis than those with the latter two subtypes (Kandoth et al., 2013).

As a therapeutic option for EC, surgery is primarily selected if the tumor is locally confined. Surgery is followed by adjuvant radiotherapy or chemotherapy for high-risk patients, such as those suffering from recurrent and metastatic disease, Stage III–IV disease, or type II EC. In these cases, cisplatin, carboplatin, and paclitaxel are the representative agents (Morice et al., 2016). Although molecular targeted therapies, including anti-angiogenesis agents and immune checkpoint inhibitors, are effective in a subset of patients with advanced or recurrent disease (Lee, 2021), treatment options for EC are still limited, underscoring the need for the development of new therapeutic options. Cancer models that accurately mimic the biological properties of human tumors are essential for efficient drug discovery and elucidation of the mechanisms of carcinogenesis. To this end, various murine *in vivo* models for EC have been established using the genetically engineered mouse (GEM) approach (Van Nyen et al., 2018). Earlier studies have mainly investigated the effect of *Pten* deletion, while the relevance of other genetic alterations remains to be elucidated.

In this review article, we review the status of genetic models for EC. Specifically, we stratified GEMs by assessing a series of *Cre* transgenic mice generated to achieve uterine-specific genetic engineering. We also illustrate recent advances in the development of cell-based *ex vivo/in vivo* hybrid models for EC and provide future perspectives in EC modeling.

## *In Vivo* Mouse Models of EC

### Background

The anatomy and physiological function of the female reproductive tract (FRT) differ significantly between humans and mice. In humans, the uterus is a single-lumen organ located in the middle of the pelvis. The two oviducts, also known as fallopian tubes (FTs), protrude from both sides of the uterus and are functionally connected with the ovaries *via* the infundibulum and fimbriae. In mice, the uterus consists of two uterine horns, and the oviducts are coiled under them inconspicuously. In humans, the reproductive cycle, called the menstrual cycle, lasts for approximately 28 days. The endometrium undergoes cyclical growth, differentiation, and shedding during that period, under the regulation of estrogen and progesterone. In contrast, this cycle in mice, called the estrous cycle, lasts approximately 4–5 days, comprising proestrus, estrus, metestrus, and diestrus periods, without the massive shedding of endometrial tissues into the lumen. Therefore, it is vital to consider these inherent inter-species differences when interpreting the phenotypes of FRT in mouse models.

The generation of GEMs has been the gold standard for modeling tumor development in a physiological setting. Cre-LoxP technology enables the spatiotemporal regulation of gene recombination, further extending its application to the fine-tuning of carcinogenesis models (Deng, 2014; Kim et al., 2018). However, there are some drawbacks to the GEM approach. First, time-consuming and labor-intensive work is often required, especially when conditional alleles are necessary for multiple genes. Second, most current *Cre* transgenic lines provide limited tissue specificity in generating EC models by intercrossing GEM with conditional floxed alleles. To develop an ideal *Cre* transgenic line, identifying genes specifically transcribed in endometrial cells is required. Despite intensive efforts, research in the field has not come up with such long-sought mice until recently.

### Mice With Systemic Genetic Engineering

*Pten*-null mice die during embryogenesis, clearly indicating their requirement for normal development. *Pten* ^+/−^ mice are born healthy but develop precancerous lesions or malignant neoplasms in multiple organs, including the endometrium, thyroid, and breast (Podsypanina et al., 1999; Stambolic et al., 2000). All *Pten* ^+/−^ mice developed endometrial hyperplasia with various degrees of atypia, 22% of which eventually progressed to EC within a year. Notably, the tumors exhibited loss of heterozygosity (LOH) at the *Pten* locus, confirming the tumor suppressor role of *Pten* in EC development. Consistent with the high frequency of MSI in EC (Black et al., 2006; Bonneville et al., 2017), the concurrent *Mlh1* loss in *Pten* ^+/−^ mice accelerates endometrial tumorigenesis, highlighting the critical role of DNA mismatch repair deficiency in EC development (Wang et al., 2002). To recapitulate the situation in middle-aged women with unopposed estrogen, 17β-estradiol pellets were implanted in *Pten* ^+/−^ mice that underwent oophorectomy. As predicted, this treatment resulted in increased cancer incidence, underscoring the pro-tumorigenic effects of estrogen. In contrast, *Pten* ^+/−^; *ERα* ^*−/−*^ mice paradoxically showed a higher incidence of *in situ* and invasive carcinoma than *Pten* ^+/−^ mice, suggesting that loss of the ERα could promote EC development. These opposing observations suggest that the effects of *Pten* inactivation and the estrogen/ERα axis might exert context-dependent effects in the development of EC, pointing toward a narrow window for achieving the right tumorigenic effects (Joshi et al., 2012). For genes other than *Pten*, there is little information on systemic GEMs with EC development. Therefore, whether a certain combination of systemic genetic alterations could lead to preferential tumor development in the uterus remains largely unknown.

### Mice With Uterine-Specific Genetic Engineering

#### Introduction

Various *Cre* transgenic mice have been generated to achieve gene recombination in the uterus (Table 1), with varying magnitudes of tissue-specific *Cre* expression. Nonetheless, certain combinations of genetic aberrations are predisposed to EC development. We illustrate representative conditional GEM models for ECs sorted by *Cre* transgenic mouse lines (Table 2).

**TABLE 1:** Transgenic Cre mice used in generating genetically engineered mice for endometrial cancer models.

	Cell-specificity in uterus		Other FRT		Main non-FRT	Reference
	Epithelium	Mesenchyme	Ovary	FT		
*Pgr-Cre*	✓	✓	✓	✓	pituitary gland, mammary gland	Soyal et al. (2005)
*Sprr2f-Cre*	✓		✓	✓	kidney, cerebellum	Contreas et al. (2010)
BAC*-Sprr2f-Cre*	✓					Cuevas et al. (2019)
*Ltf-iCre*	✓			✓	mammary gland, neutrophil	Daikoku et al. (2014)
*Ksp.1.3-Cre*	✓			✓	kidney	Shao et al. (2002)
*Pax8-*rtTA*;* TetO*-Cre* ^a^	✓			✓	kidney, hepatocyte	Perets et al. (2013)
CAG*-Cre-ER* ^*T*^ ^b^	✓		N.E.	N.E.	lung, colon, kidney, thyroid, liver	Hayashi and McMahon (2002)
*Amhr2-Cre*		✓	✓	✓		Jamin et al. (2002)
Adenovirus*-Cre*	✓	✓^c^				Beauparlant et al. (2004)

FRT, female reproductive tract; N.E., not examined.

a Drinking water containing doxycyclin.

b Intraperitoneal injection of tamoxifen.

c Low penetration.

**TABLE 2:** Past literature on genetically engineered mice for endometrial cancer models.

Cre line	Targeted gene	Penetrance	Latency	EC type	Major histological type	Reference
Systemic	*Pten* ^*+/−*^	100%	18–39w	-	CAH	Podsypanina et al. (1999)
	*Pten* ^*+/−*^	21%	30–66w	I	AC (poorly diff.)	Stambolic et al. (2000)
	*Pten* ^*+/−*^ *; Mlh1* ^*−/−*^	40%	14–18w	I	AC (well diff.)	Wang et al. (2002)
	*Pten* ^*+/−*^ *; ER* ^*−/−*^	25%	32w	I	AC	Joshi et al. (2012)
*Pgr-Cre*	*Pten* ^*fl/fl*^	89%	12w	I	AC	Daikoku et al. (2008)
	*Pten* ^*fl/fl*^ *; Kras* ^*LSL-G12D/+*^	100%	4w	I	AC (EMC)	Kim et al. (2010)
	*Pten* ^*fl/fl*^ *; Tgfbr1* ^*fl/fl*^	100%	16w	I	AC	Gao et al. (2017)
	*Tgfbr1* ^*fl/fl*^	N.A.	N.A.	I	AC	Monsivais et al. (2019)
	*Smad2* ^*fl/fl*^ *; Smad3* ^*fl/fl*^	100%	34w	I	AC (EMC)	Kriseman et al. (2019)
	*Cdh1* ^*fl/fl*^ *; Trp53* ^*fl/fl*^	47%	48w	II	AC	Stodden et al. (2015)
	*Pten* ^*fl/fl*^ *; Dicer1* ^*fl/fl*^	100%	12w	II	AC (poorly diff.)	Wang et al. (2020)
*Sprr2f-Cre*	*Lkb1* ^*fl/fl*^	100%	30w	I	AC	Contreas et al. (2010)
	*Lkb1* ^*fl/fl*^ *; Ccl2* ^*−/−*^	100%	43–58w	I	AC	Peña et al. (2015)
	*Trp53* ^*fl/fl*^ *; Pot1a* ^*fl/fl*^	100%	60w	II	AC	Akbay et al. (2013)
BAC*-Sprr2f-Cre*	*Pten* ^*fl/fl*^ *; Fbxw7* ^*fl/fl*^	100%	40–67w	II	CS	Cuevas et al. (2019)
	*Pole* ^*LSL-P286R/+*^ *; Msh2* ^*fl/fl*^	100%	45w	I	AC (EMC)	Li et al. (2020)
*Ltf-iCre*	*Pten* ^*fl/fl*^	100%	12w	-	CAH	Liang et al. (2018)
	*R26-Pik3ca* ^*H1047R*^ *; Arid1a* ^*fl/fl*^	100%	20w	I	AC (moderately to poorly diff.)	Wilson et al. (2019)
*Ksp1.3-Cre*	*Trp53* ^*fl/fl*^	84%	65–79w	II	AC (SC, CCC), CS, Undiff. Ca	Wild et al. (2012)
*Pax8-*rtTA*;* TetO*-Cre*	*Pten* ^*fl/fl*^ *; Arid1a* ^*fl/fl*^	100%	6–12w	I	AC (EMC)	Suryo Rahmanto et al. (2020)
	*Trp53* ^*fl/fl*^ *; Rb1* ^*fl/fl*^	81%	57w	II	AC (SC)	Fu et al. (2020)
CAG*-Cre-ER* ^*T*^	*Pten* ^*fl/fl*^	100%	6–8w	-	AC *in situ*	Mirantes et al. (2013)
*Amhr2-Cre*	*Pten* ^*fl/fl*^ *; Kras* ^*LSL-G12V/+*^	90%	22–56w	-	Stromal tumor	Kun et al. (2020)
Adenovirus*-Cre*	*Pten* ^*fl/fl*^	41%	16–32w	I	AC	Joshi and Ellenson (2011)
	*Pten* ^*fl/fl*^	88%	2w	I	CAH, AC (EMC)	Saito et al. (2011)
	*Pten* ^*fl/fl*^ *; Lkb1* ^*fl/fl*^	100%	8–28w	I	AC (EMC)	Cheng et al. (2014)
	*Pten* ^*fl/fl*^ *; Kras* ^*LSL-G12D/+*^	50%	14w	I	AC (EMC)	Tirodkar et al. (2014)

AC, adenocarcinoma; CAH, complex atypical hyperplasia; CCC, clear cell carcinoma; CS, carcinosarcoma; EMC, endometrioid carcinoma; SC, serous carcinoma; diff., differentiated; Undiff. Ca, undifferentiated carcinoma; N.A., not available.

#### *Pgr-Cre* Mice

The progesterone receptor (*Pgr*) is expressed in the uterus and widely across the FRT. Transgenic *Pgr-Cre* mice expressing *Cre* under the control of the *Pgr* promoter (Soyal et al., 2005) have been most commonly used in the generation of GEM for EC models, despite its incomplete tissue specificity (Table 1). As *Pgr* expression is observed in all major types of cells in the uterus, including epithelium, myometrium, and stroma, floxed genes are deleted in both epithelial and non-epithelial components of the uterus in *Pgr-Cre* mice. Therefore, genetic aberrations are not recapitulated exclusively in endometrial cells in mice, unlike in sporadic cases of EC in humans. *Pgr-Cre*; *Pten* ^*fl/fl*^ mice frequently develop invasive type I EC by 3 months of age (Daikoku et al., 2008), suggesting that the endometrium is most susceptible to tumorigenesis by *Pten* inactivation in FRT organs. *Kras* ^*G12D*^ accelerates *Pten* inactivation-dependent EC development, underscoring the oncogenic role of mutant *Kras* in the endometrium (Kim et al., 2010). In the pathogenesis of human cancers, the transforming growth factor β (TGFβ) signaling pathway has multifaceted roles, either pro- or anti-tumorigenic, depending on the organ and genetic context (Gordon and Blobe, 2008). Consistent with this notion, *Pgr-Cre*; *Tgfbr1* ^*fl/fl*^; *Pten* ^*fl/fl*^ mice develop metastatic EC (Gao et al., 2017). It was later demonstrated that even *Pgr-Cre*; *Tgfbr1* ^*fl/fl*^ mice develop estrogen-dependent ECs with lung metastasis (Monsivais et al., 2019). Furthermore, *Pgr-Cre*; *Smad2* ^*fl/fl*^; *Smad3* ^*fl/fl*^ mice develop endometrial hyperplasia, which eventually progresses to bulky endometrioid carcinoma with complete mortality by 8 months of age (Kriseman et al., 2019). Based on these results, it is likely that inactivation of the TGFβ signaling pathway plays a pro-tumorigenic role, at least in the pathogenesis of type I ECs. Some GEMs that recapitulate type II EC have also been established. *TP53* mutation and *CDH1* inactivation are common in type II EC (Samarnthai et al., 2010). Consistent with this finding, *Pgr-Cre*; *Cdh1* ^*fl/fl*^; *Trp53* ^*fl/fl*^ mice develop typical type II EC at 6 months of age, with histological features including papillary growth, hobnailing, and severe nuclear atypia (Stodden et al., 2015). In addition, metastasis to nearby and distant organs within the peritoneal cavity was evident at 12 months of age. Intriguingly, these tumors are characterized by highly inflammatory microenvironments with prominent immune cell recruitment. Consistent with the notion *that DICER1* is a putative tumor suppressor gene in EC (Bailey et al., 2018), *Pgr-Cre*; *Dicer1* ^*fl/fl*^; *Pten* ^*fl/fl*^ mice develop hormone-independent poorly differentiated EC that is positive for clear cell carcinoma markers, such as napsin A and hepatocyte nuclear factor (HNF) 1β (Wang et al., 2020).

#### *Sprr2f-Cre* Mice

*Sprr2f* is exclusively expressed in the uterus, particularly in endometrial epithelial cells (Contreras et al., 2010). Hence, it may be an ideal candidate for a gene promoter used in *Cre* transgenic mice for EC development. To this end, *Sprr2f-Cre* transgenic mice were generated by fusing a 5.8 kb *Sprr2f* promoter fragment with *Cre*. Despite uterus-specific expression of endogenous *Sprr2f*, *Cre* activity in this mouse was observed not only in the endometrium but also in the kidney and cerebellum (Contreras et al., 2010), as demonstrated by the *ROSA26* reporter knock-in mice (R26R) (Soriano, 1999). This ectopic leaky expression in *Sprr2f-Cre* mice might be attributed to the short length of the promoter fragment, which could lack distant regulatory elements required for the repression of its non-endometrial expression. Furthermore, gene recombination in the adult uterine epithelia exhibited a mosaic pattern. Thus, there might be room for further refinement of these *Sprr2f-Cre* mice. Nevertheless, *Sprr2f-Cre*; *Lkb1* ^*fl/fl*^ mice develop invasive ECs and eventually die by 30 weeks of age (Contreras et al., 2010). Concurrent deletion of *cytokine chemokine ligand 2* (*Ccl2*) significantly attenuates *Lkb1* loss-driven tumor progression, revealing the involvement of inflammation in EC development (Peña et al., 2015). The development of type II EC has been associated with telomeric dysfunction (Akbay et al., 2008). Consistent with this notion, the concurrent deletion of *Trp53* and *Pot1a*, which encodes a component of the shelterin complex that stabilizes telomeres, in *Sprr2f-Cre*; *Pot1a* ^*fl/fl*^; *Trp53* ^*fl/fl*^ mice leads to the development of type II EC (Akbay et al., 2013).

#### *BAC-Sprr2f-Cre* Mice

To recapitulate the highly endometrium-specific transcriptional regulation of *Sprr2f* in mice, the bacterial artificial chromosome (BAC) clone RP23-3914 was introduced to drive *Cre* expression instead of the shorter promoter sequence used in *Sprr2f-Cre* mice. Since the BAC clone harbors a 189 kb genomic fragment spanning the *Sprr2* tandem gene cluster region on chromosome 3, it is postulated to be long enough to recapitulate all endogenous transcriptional regulation accurately. Indeed, BAC*-Sprr2f-Cre* transgenic mice allowed gene recombination in a highly endometrium-specific manner. EC has the highest incidence of *FBXW7* mutations among all human cancers, followed by colon cancer (Yeh et al., 2018). BAC*-Sprr2f-Cre*; *Pten* ^*fl/fl*^; *Fbxw7* ^*fl/fl*^ mice developed endometrioid carcinoma, which eventually progressed to CS at 40–67 weeks. Genomic analysis revealed that most tumors spontaneously acquired the *Trp53* mutation, in line with the critical role of p53 loss in CS development (Cuevas et al., 2019). Thus, this GEM represents a unique model that exhibits a transition from type I to type II EC. *POLE* encodes a DNA polymerase epsilon, mutated in 7–12% of ECs, and the P286R mutation drives an ultra-mutator phenotype (Kandoth et al., 2013; Rayner et al., 2016). BAC*-Sprr2f-Cre*; *Pole* ^*P286R/+*^ mice developed EC with complete penetrance, which exhibited mutation signatures similar to human EC, while remaining relatively stable in gene copy number. These findings are consistent with those of POLE-type ultra-mutated EC. Further tumor progression is achieved by concurrent *Msh2* deletion (Li et al., 2020).

#### *Ltf-iCre* Mice

Lactoferrin (Ltf), a non-heme iron-binding glycoprotein, is highly expressed in the uterine epithelium of adult mice in response to estrogen exposure (McMaster et al., 1992; Teng et al., 2002). *Ltf-improved Cre* (*iCre*) transgenic mice were generated as an endometrium-specific *iCre* mouse line. However, ectopic expression was observed in mammary glands and neutrophils (Daikoku et al., 2014). *iCre* is a modified *Cre* gene that reduces the high CpG content of the prokaryotic coding sequence, thereby reducing the chances of epigenetic silencing in mammals (Shimshek et al., 2002). Whereas *Pgr-Cre*; *Pten* ^*fl/fl*^ mice develop EC with high penetration (Daikoku et al., 2008), *Ltf-iCre*; *Pten* ^*fl/fl*^ mice develop uterine complex atypical hyperplasia, but not carcinoma (Liang et al., 2018). These results suggest that *Pten* inactivation in the uterine stroma can promote the transformation of *Pten*-null epithelial cells, which may not be potently tumorigenic on their own. Considering that *Pten* inactivation in the uterine stroma is observed in Cowden’s disease, but not common in sporadic cases of human EC, these results suggest that *Pgr-Cre*; *Pten* ^*fl/fl*^ mice and *Ltf-iCre*; *Pten* ^*fl/fl*^ mice may mimic hereditary and sporadic cases of EC, respectively. *ARID1A* loss frequently co-occurs with mutations in genes involved in the PI3K pathway in ECs (Liang et al., 2012). Consistent with this finding, *Pgr-Cre*; R26-*Pik3ca* ^*H1047R*^; *Arid1a* ^*fl/+*^ mice develop ECs (Wilson et al., 2019), suggesting that *ARID1A* is a haploinsufficient tumor suppressor in EC development.

#### *Ksp1.3-Cre* Mice

*Ksp-1.3* (also known as *Cdh16*) is expressed exclusively in the adult kidney and developing genitourinary tract in mice (Thomson et al., 1995; Thomson et al., 1998). *Ksp1.3-Cre* activity is usually observed in a mosaic pattern in endometrial epithelial cells of the lumen and glands (Shao et al., 2002; Frew et al., 2008). *Ksp1.3-Cre*; *Trp53* ^*fl/fl*^ mice develop type II ECs, such as serous carcinoma, clear cell carcinoma, and CS, in 84% of cases at 58–68 weeks of age (Wild et al., 2012). In these tumors, the mTORC1 signaling pathway is frequently activated in precancerous lesions and tumors, suggesting that its cooperation with *Trp53* loss leads to the development of type II EC.

#### *Pax8-*rtTA; TetO*-Cre* Mice

Pax8 is a lineage-specific transcription factor that marks the Müllerian lineage epithelium, such as the uterus and FT, but not the ovary (Mittag et al., 2007). The *Pax8-*rtTA; TetO*-Cre* mouse contains a reverse tetracycline (Tet)-regulated transactivator under the control of the murine *Pax8* promoter and TetO*-Cre*, thereby enabling gene recombination in an inducible manner by doxycycline administration. Using this mouse model, the effects of various combinations of conditional alleles of *Brca1/2*, *Trp53*, and *Pten* were investigated. While these mice predominantly developed high-grade serous carcinoma from secretory epithelial cells in the FT, various degrees of endometrial lesions were also induced to a lesser extent, resembling endometrial hyperplasia, dysplasia, and carcinoma in humans (Perets et al., 2013). Because mutations in *ARID1A* and *PTEN* frequently co-occur in uterine endometrioid carcinoma, *Pax8-*rtTA; TetO*-Cre*; *Pten* ^*fl/fl*^; *Arid1a* ^*fl/fl*^ mice were generated. Six weeks after doxycycline administration, these mice developed gross uterine tumors accompanied by local and peritoneal dissemination. Intriguingly, induced tumors exhibited histological features of endometrioid carcinoma and displayed similar gene expression profiles to those in human endometrioid carcinoma. Loss of either gene alone did not induce any gross tumor (Suryo Rahmanto et al., 2020), which may be indicative of cooperation between the losses of *ARID1A* and *PTEN* in EC development. The development of EC with histological features of serous carcinoma was observed in 81% of *Pax8-*rtTA; TetO*-Cre*; *Trp53* ^*fl/fl*^; *Rb* ^*fl/fl*^ mice (Fu et al., 2020).

#### CAG*-Cre-ER* ^*T*^ Mice

The CAG promoter is a hybrid construction of the cytomegalovirus (C) early enhancer element fused to the chicken β-actin (A) gene promoter and the splice acceptor of the rabbit β-globin (G) gene. Therefore, it is supposed to function as a promoter that drives high levels of transcription in most cells and tissues. However, in the CAG*-Cre-ER* ^*T*^ transgenic mouse, in which Cre expression is induced by tamoxifen administration (Hayashi and McMahon, 2002), intraperitoneal (i.p.) injection of tamoxifen leads to gene recombination mainly in epithelial cells throughout the body in a dose-dependent manner. After i.p. injection of tamoxifen, CAG*-Cre-ER* ^*T*^; *Pten* ^*fl/fl*^ mice developed endometrial hyperplasia and adenocarcinoma *in situ*, while thyroid hyperplasia was also ectopically observed (Mirantes et al., 2013). These results underscore the higher susceptibility of the endometrium to *Pten* loss-dependent tumorigenesis compared to any other organ. However, considering the ubiquitous expression pattern, it remains to be seen whether transgenic CAG-*Cre-ER* ^*T*^ mice are also useful in EC development driven by other genetic alterations.

#### *Amhr2-Cre* Mice

*Amhr2* is expressed in the Müllerian duct mesenchyme and the adjacent mesonephric epithelium (Arango et al., 2008). *Amhr2-Cre* mice undergo gene recombination in the uterus, selectively in the cells of the stroma and myometrium, but not in the epithelium (Jamin et al., 2002; Daikoku et al., 2013). *Amhr2-Cre*; *Pten* ^*fl/fl*^ mice fail to develop EC, but instead potently induced the conversion of myometrial cells into adipocytes (Daikoku et al., 2011). *Amhr2-Cre*; *Pten* ^*fl/fl*^; *Kras* ^*LSL-G12V/+*^ mice develop uterine stromal tumors, such as leiomyoma and leiomyosarcoma, but not epithelial tumors (Kun et al., 2020). These findings suggest that *Amhr2-Cre* mice may not be suitable for uterine epithelial tumorigenesis.

#### Intrauterine Injection of Adenovirus*-Cre*

As an alternative approach to achieve conditional gene recombination in the endometrium of adult mice, local injection of adenovirus encoding *Cre* (Adeno-*Cre*) has been adopted (Beauparlant et al., 2004). After the intrauterine (i.u.) injection of Adeno-*Cre*, *Pten* ^*fl/fl*^ mice developed EC, albeit with partial penetrance (Joshi and Ellenson, 2011; Saito et al., 2011). After Adeno-*Cre* injection*, Pten* ^*fl/fl*^; *Kras* ^*LSL-G12D/+*^ mice and *Pten* ^*fl/fl*^; *Lkb1* ^*fl/fl*^ mice developed EC with 50% penetrance (Tirodkar et al., 2014) and 100% penetrance (Cheng et al., 2014), respectively. These results suggest that *Lkb1* inactivation cooperates more profoundly than *Kras* activation. However, both genetic alterations are thought to activate the PI3K pathway. Visualization of gene recombination using R26R mice demonstrated that gene recombination by the i.u., injection of Adeno-*Cre* is not completely epithelium-specific or uniformly achieved in targeted cells (Joshi and Ellenson, 2011). Nonetheless, it seems likely that the Adeno-*Cre* approach has been successful, at least for modeling EC development driven by *Pten* deletion*.*

## *Ex Vivo/In Vivo* Hybrid Mouse Models for EC

### General Overview

In addition to pursuing tissue specificity *in vivo* by developing novel *Cre* transgenic mice with the promoter of endometrium-specific genes, researchers have also attempted to achieve the same goal using an alternative approach. It is a cell-based carcinogenesis model comprising two steps: *1*) *ex vivo* culture of murine primary endometrial cells followed by gene transduction to reconstitute EC-specific mutation and *2*) allograft implantation of transduced cells into immunocompromised mice for *in vivo* monitoring of tumor development. An obvious advantage of this *ex vivo/in vivo* hybrid approach is that the cell and tissue type specificity is warranted by physical separation of the uterus and that the outcome is apparent within a short period. On the other hand, the caveat is that the authentic uterine microenvironment, including the proficient immune response, is not thoroughly provided, potentially affecting the outcome depending on the genetic alterations tested in this model. At any rate, no model is perfect by itself. Ideally, they should complement one another to move EC research forward. Below, we have illustrated recent efforts, including ours, to develop cell-based *ex vivo/in vivo* hybrid models for endometrial carcinogenesis.

### Monolayer Cell-Based Model

To achieve uterine-specific genetic engineering *ex vivo*, the uterus was isolated from wild-type (WT) mice. After enzymatic and mechanical dissociation, primary murine epithelial cells were transiently prepared and expanded in a Petri dish. Genetic engineering, such as the introduction of *myr*-*Akt* (encoding a membrane-bound constitutive active form of Akt) or sh*Pten* (encoding short hairpin RNA against *Pten*) into primary endometrial cells, is conducted using lentiviral vectors. By implanting transduced endometrial cells, together with the WT uterine stromal cells from neonates, into the renal capsule of severe combined immunodeficiency (SCID) mice, adenocarcinoma development was achieved (Memarzadeh et al., 2010). This study demonstrated that the generation of GEM is dispensable for the induction of EC in murine cells. Similarly, epithelial cells isolated from the uterus of GEM also develop tumors. After isolation of the uterus from *Pten* ^*fl/fl*^ mice, epithelial cells were separated from cultured stromal cells using flow cytometry. After infecting these cells with lentivirus encoding *Cre* (LV-*Cre*), the resultant *Pten*-null epithelial cells and WT uterine stroma were jointly implanted into the renal capsule of oophorectomized SCID mice. With estrogen supplementation, adenocarcinoma-like tumors eventually develop (Janzen et al., 2013). These two studies suggest the importance of cooperation between epithelia and stroma for the development of *Pten* deletion-driven EC, although whether co-transplanted uterine stromal cells are dispensable for tumor development is yet to be investigated. In addition, it remains to be seen whether tumors can develop even in the subcutis, where there is a lower blood supply.

### Organoid-Based Model

#### General Overview

**FIGURE 1:**
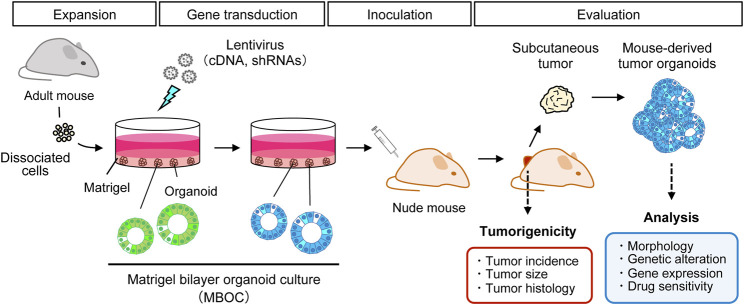
Organoid-based *ex vivo/in vivo* hybrid carcinogenesis model. Primary cells were isolated and subjected to the Matrigel bilayer organoid culture method. Cancer-specific recurrent genetic alterations are reconstituted in murine organoids by lentiviral infection. These transduced organoids were inoculated into the subcutis of nude mice to monitor tumor development for 8–10 weeks. The effects of particular genetic alterations on tumorigenic potential were evaluated in terms of the incidence, size, and histology of the tumors. Induced subcutaneous tumors and mouse-derived tumor organoids can be analyzed using many downstream assays.

Organoid culture is an emerging technology that enables the self-renewal and differentiation of normal epithelial stem cells in a matrix-assisted three-dimensional structure supplemented with distinct stem cell niche factors. As organoids are maintained in serum-free media, epithelial cells are selectively propagated in response to EGF, while non-epithelial cells are quickly and automatically depleted from the culture. Thus, flow cytometry-based sorting is not necessary for the purification of epithelial cells. It was first demonstrated that murine intestinal stem cells marked by *Lgr5* could infinitely proliferate *in vitro*, giving rise to all differentiated lineages of the small intestine (Sato et al., 2009). Organoid culture technology has been extensively applied to various cancer research fields, including carcinogenesis, drug resistance, and drug discovery (Drost and Clevers, 2018). We previously developed an efficient *in vitro* gene transduction method using a lentivirus (Maru et al., 2016), which is based on the Matrigel bilayer organoid culture (MBOC) protocol (Maru et al., 2019a). This technical innovation enabled us to establish the first murine organoid-based carcinogenesis model with WT normal cells in the *ex vivo/in vivo* setting (Figure 1). It recapitulated multi-step colon carcinogenesis in the subcutis of nude mice without generating GEM. Similarly, multiple genetic alterations were reconstituted in murine and human intestinal organoids using CRISPR/Cas9 technology to generate organoid models of multi-step colorectal carcinogenesis (Drost et al., 2015; O'rourke et al., 2017). Although tumor development from murine organoids is achieved by one or two genetic alterations, it remains technically challenging to induce full-blown tumors from normal human organoids even with three to four genetic alterations. This suggests that murine cells might have a lower threshold for tumorigenesis, presumably reflecting a shorter lifespan in mice.

Using this approach, we demonstrated that reconstitution of the common genetic alterations in each organ could considerably and generally recapitulate multi-step carcinogenesis independently of the tissue-specific microenvironment. For example, mutant *Kras* in intestinal organoids markedly accelerates tumorigenesis caused by *Apc* knockdown alone (Onuma et al., 2013). In organoids from the hepatobiliary tract (Ochiai et al., 2019) and pancreas (Matsuura et al., 2020), full-blown tumors are developed not by mutant *Kras* alone but by concurrent inactivation of the p53 or Rb pathways. These outcomes are consistent with the results from earlier *in vivo* GEM studies with identical genetic alterations, if the tumorigenic potential was evaluated based on the tumor development rate, size, and histology. Therefore, these findings point toward the notion that these models could, at least partly, substitute and complement GEMs in evaluating the pathological relevance of reconstituted genetic interactions. *Ex vivo/in vivo* hybrid carcinogenesis models using murine FT organoids have been recently described (Maru et al., 2021b). Although *Trp53* loss alone is not sufficient for tumor development in FT organoids, the concurrent introduction of common genetic aberrations in ovarian high-grade serous carcinoma leads to the development of tumors of various grades and histological features. For example, adding *Pik3ca* ^*H1047R*^ or *Kras* ^*G12D*^ to p53-null FT organoids leads to the development of adenocarcinoma and CS, respectively. Drug sensitivity varies among mouse-derived tumor organoids (MDTO) with different genetic aberrations.

Given the ectopic nature as a tumor developing site and the lack of the right immune response, the subcutis of Balb/c background nude mice may not be ideal for C57bl/6J background endometrial organoids for tumor development. However, we initially did not pay much attention to this situation because the purpose of inoculating transduced organoids in nude mice was to detect cellular transformation, which was supposed to be achieved by genetic engineering alone. Intriguingly, a detailed analysis of MDTO revealed that this artificial setting supported *Kras*-driven carcinogenesis from pancreatic organoids in a way that recapitulates pancreatic carcinogenesis in humans (Matsuura et al., 2020). For example, organoids acquired genetic or epigenetic changes after subcutaneous inoculation, which were, in turn, positively selected by nude mice for more malignant tumors. Moreover, mutation signatures in MDTO were similar to those observed in human pancreatic cancer. Based on these findings, it is tempting to speculate that the hybrid carcinogenesis models might depend on both *ex vivo* and *in vivo* parts. Both would further cooperate for tumorigenesis. Thus, we updated our view on *ex vivo/in vivo* hybrid carcinogenesis models that had initially emphasized the *ex vivo* part.

#### Endometrial Organoid-Based Model

Murine endometrial organoids recapitulate the physiological response of the endometrial epithelium to hormones, including estrogen and progesterone (Boretto et al., 2017). However, it remains unclear whether carcinogenesis could also be recapitulated. We recently aimed to establish an *ex vivo/in vivo* hybrid carcinogenesis model using endometrial organoids (Maru et al., 2021a). Unlike in earlier *in vivo* studies using GEM, inactivation of *Pten* in endometrial organoids has only a marginal impact on tumorigenesis, even when combined with mutant *Kras*. These results reconfirmed the relevance of the uterine stroma in endometrial carcinogenesis. We are currently testing many other combinations of genetic alterations that could potentially cooperate for EC development. We have already generated multiple tumors, and detailed information will be reported elsewhere.

Intriguingly, the propagation of endometrial organoids was unexpectedly halted after the lentiviral introduction of the pLKO.1-puro vector and sh*Luc* (shRNA against *luciferase*), an empty backbone vector and the negative control shRNA, respectively (Maru et al., 2021a). These phenomena have never been observed in any other tissue-derived organoids under almost the same culture conditions (Onuma et al., 2013; Ochiai et al., 2019; Matsuura et al., 2020; Maru et al., 2021b). In addition, endometrial organoids with potentially advantageous alterations, such as *Cre*-mediated *Kras* ^*G12D*^ induction or shRNA-mediated *Pten* knockdown, continued to proliferate over many passages after lentiviral infection. Together, these observations raise the possibility that endometrial organoids may be extremely sensitive to DNA damage triggered by genome integration following lentiviral infection, which could be overcome by oncogenic signals. Alternatively, this might imply that the currently adopted culture conditions require further optimization for endometrial organoids.

Notably, *Cdkn2a* knockdown or *Trp53* deletion in *Kras* ^*G12D*^-expressing endometrial organoids leads to CS or monophasic sarcoma development *via* extensive epithelial-mesenchymal transition (Maru et al., 2021a). Re-inoculating MDTO into the subcutis of nude mice revealed that the transition from carcinoma to CS might be unidirectional. Considering that identical genetic aberrations in pancreatobiliary organoids always result in the development of adenocarcinoma (Ochiai et al., 2019; Matsuura et al., 2020), these findings, together with the results of a study with FT organoids, suggest that gynecological organs, in general, may be predisposed to CS due to some inherent epigenetic status. Detailed examination of organoids in each step of the models revealed that spontaneous deletion of the *Kras* ^*WT*^ allele frequently occurs in MDTOs with *Kras* ^*G12D*^ and sh*Cdkn2a* knockdown, but not in those with *Kras* ^*G12D*^ and *Trp53* deletion. These findings are in line with the notion that the *Kras* ^*WT*^, which is normally regarded as an oncogene, serves as a relative tumor suppressor gene in the presence of *Kras* ^*G12D*^ (Zhang et al., 2001), suggesting that *Cdkn2a* suppression might be less tumorigenic than *Trp53* loss in the context of *Kras*-driven tumorigenesis in gynecological organs. It remains to be seen whether tumor development can be achieved in GEM models with identical genetic alterations.

## Discussion

The development of genetic mouse models for EC has been extended in two different directions. One conventional method is to pursue the generation of GEMs with the highest specificity for genetic engineering of the uterine epithelium. To this end, the two *Cre* lines, *Ltf-iCre* (Daikoku et al., 2014) and BAC*-Sprr2f-Cre* (Cuevas et al., 2019), are currently the most sophisticated in terms of accurate genetic engineering of the endometrium. As the cooperation between epithelial cells and the microenvironment, such as stromal cells and immune cells, is also integrated, these *in vivo* GEM models will likely contribute to the elucidation of the molecular mechanisms underlying EC initiation and progression. Hence, it is unlikely that there is a growing demand for the development of novel superior *Cre* lines in generating EC models. Researchers would rather generate more GEMs with diverse combinations of genetic alterations using these available *Cre* lines. Most previous studies mainly focused on histological features but not genomic, transcriptomic or proteomic features concerning the similarity between induced mouse uterine tumors and human EC. Now that public databases of so-called Omics-based analysis of human tumors have become available, future studies will definitely need to include these data in assessing the inter-species similarity of the tumors.

Another new way is to develop *ex vivo/in vivo* hybrid models based on genetic engineering of primary endometrial cells or organoids. Given the rapid application of organoids and CRISPR/Cas9 technology in many research fields (Izumiya et al., 2021), it is probable that an increasing number of organoid-based models for EC will be developed. However, only a few such models have been documented for FRT organs to date (Zhang et al., 2019; Lohmussaar et al., 2020; Maru et al., 2021a; Maru et al., 2021b), including ours being the first and only EC model (Maru et al., 2021a). Although this study described the development of a novel model for uterine CS, it belongs to a minor category of type II EC, and no organoid-based model for type I EC has been developed so far. However, in an ongoing project, we are developing new organoid-based type I EC models. These results will also shed light on the mechanisms by which the outcomes could vary between *in vivo* and *ex vivo/in vivo* hybrid models for EC.

One of the obvious shortcomings of the current *ex vivo/in vivo* hybrid carcinogenesis model is the absence of a proficient immune response and physiological tissue-specific microenvironment. To overcome this limitation associated with the subcutis of nude mice, we recently established an orthotopic gallbladder (GB) cancer model using syngeneic mice (Kato et al., 2021). Specifically, we generated genetically engineered organoids *in vitro* using LV-*Cre* and CRISPR/Cas9 technology. The organoids were inoculated into the dorsal skin of a syngeneic WT mouse, which developed subcutaneous tumors in several weeks. Subsequently, minced tumor fragments were directly sutured to the outer surface of the GB of another syngeneic WT mouse. This novel two-step model enabled detailed analysis of tumor-infiltrating immune cells and evaluation of drug responses in more physiological settings. Notably, the therapeutic effects of immune checkpoint inhibitors will become feasible.

In our earlier studies, the subcutis of nude mice was selected as the target site for organoid inoculation. Still, it was mainly for technical convenience and did not necessarily mean using nude mice is required. Indeed, we confirmed that transduced pancreatic organoids could develop in the subcutis of syngeneic mice, albeit smaller in size (Matsuura et al., 2020). Moreover, orthotopic implantation of or tumor organoids or fragments derived from nude mice efficiently developed tumors in the pancreas. Similarly, we obtained subcutaneous tumors in syngeneic mice with a subset of endometrial organoids expressing *Kras* ^*G12D*^ and shRNAs targeting certain tumor suppressor genes. Therefore, the application of the abovementioned two-step approach to transduced endometrial organoids that are proven tumorigenic in nude mice might be worth testing and should be further pursued. Such efforts will probably lead to significant improvements in the current organoid-based EC model as a preclinical model.

Considering the feasibility of organoid culture of patient-derived normal endometrium and endometriosis (Nikolakopoulou and Turco, 2021), establishing an organoid-based carcinogenesis model in humans might also be plausible. Patient-derived tumor organoids (PDTO) from diverse types of cancer (Bleijs et al., 2019; Maru et al., 2019b; Maru and Hippo, 2019), including EC (Boretto et al., 2019; Maru et al., 2019c; Alzamil et al., 2021), have been recently established. Collectively, integrated analyses of the two-way mouse models of EC, namely *in vivo* GEM models and *ex vivo/in vivo* hybrid carcinogenesis models, and PDTO as a human model, will probably accelerate research on EC in many aspects, such as the elucidation of the mechanisms underlying carcinogenesis and development of new therapeutic strategies.

## Concluding Remarks

Mouse genetic models that recapitulate EC development to varying extents in *in vivo* or *ex vivo/in vivo* hybrid settings have been developed. Precise modeling of EC and mutual comparison will contribute to both basic and translational research on EC. We hope that this review article will help researchers in the field to develop novel EC models.
